# Mutation of chromatin modifiers; an emerging hallmark of germinal center B-cell lymphomas

**DOI:** 10.1038/bcj.2015.89

**Published:** 2015-10-16

**Authors:** M A Lunning, M R Green

**Affiliations:** 1 Lymphoma Precision Medicine Laboratory, Dr James O Armitage Center for Leukemia and Lymphoma Research, University of Nebraska Medical Center, Omaha, NE, USA; 2 Eppley Institute for Research in Cancer and Allied Diseases, University of Nebraska Medical Center, Omaha, NE, USA; 3 Department of Internal Medicine, College of Medicine, University of Nebraska Medical Center, Omaha, NE, USA

## Abstract

Subtypes of non-Hodgkin's lymphomas align with different stages of B-cell development. Germinal center B-cell (GCB)-like diffuse large B-cell lymphoma (DLBCL), follicular lymphoma (FL) and Burkitt's lymphoma (BL) each share molecular similarities with normal GCB cells. Recent next-generation sequencing studies have gained insight into the genetic etiology of these malignancies and revealed a high frequency of mutations within genes encoding proteins that modifying chromatin. These include activating and inactivating mutations of genes that perform post-translational modification of histones and organize chromatin structure. Here, we discuss the function of histone acetyltransferases (CREBBP, EP300), histone methyltransferases (KDM2C/D, EZH2) and regulators of higher order chromatin structure (HIST1H1C/D/E, ARID1A and SMARCA4) that have been reported to be mutated in ⩾5% of DLBCL, FL or BL. Mutations of these genes are an emerging hallmark of lymphomas with GCB-cell origins, and likely represent the next generation of therapeutic targets for these malignancies.

## Introduction

Approximately 95% of lymphomas originate from B cells, the antibody-producing cells of the body. These cells develop through a complex process of differentiation, with the stages characterized by the specific structure of the B-cell receptor and the expression patterns of differentiation markers. Precursor B cells develop in the bone marrow, where they undergo a process of DNA breakage and recombination to rearrange the immunoglobulin heavy-chain and light-chain genes. Cells that produce an intact B-cell receptor that is not self-reactive are able to differentiation into mature naive B cells and enter the periphery, while the remainder die by apoptosis. Upon encounter of antigen that is recognized by their B-cell receptor, mature naive B cells become activated. For the majority of antigens, which require T-cell help for robust responses, activated B cells expand within germinal centers in secondary lymphoid organs. These germinal center B (GCB) cells are highly proliferative and edit their immunoglobulin genes via the introduction of point mutations (somatic hypermutation) and by performing further recombination to select alternative heavy-chain genes (class switch recombination). These cells can then terminally differentiate into memory B cells or antibody-producing plasma cells.

Molecular profiling studies have revealed similarities between different subtypes of non-Hodgkin's lymphomas and normal stages of B-cell differentiation (Reviewed by Kuppers^1^). This includes the alignment of three clinically and histologically distinct subtypes of lymphoma with normal GCB cells.^2^ Diffuse large B-cell lymphoma (DLBCL), the most common form of non-Hodgkin's lymphoma, can be stratified into two subtypes that transcriptionally resemble normal GCB cells (GCB-like) or post-GCB activated B cells (ABC-like).^3^ These two subtypes have unique genetic etiology, with mutations that activate the B-cell receptor signaling pathway being prevalent in the ABC-like subtype but largely absent from the GCB-like subtype (Reviewed by Pasqualucci^4^). The second most common type of non-Hodgkin's lymphoma, follicular lymphoma (FL), is named for its histologic similarities with normal lymphoid follicles and the malignant cells also resemble normal GCB cells at the molecular level.^1^ These lymphomas also share some genetic similarities to GCB-like DLBCL, and transform at a rate of 2–3% per year to a DLBCL histology. Burkitt lymphoma (BL) represents only 2% of lymphomas, and is categorized as either endemic, sporadic or immunodeficiency related. Endemic BL is driven by Epstein-Barr virus and most frequently found in areas with endemic malaria, while sporadic BL is rarely associated with Epstein-Barr virus and has no geographic bias, and immunodeficiency-related BL is primarily associated with human immunodeficiency virus infection.^5^ Sporadic BL has long been suggested to align with GCB cells,^6^ but recent next-generation sequencing studies have shown it to share less similarities in genetic etiology than those between GCB-like DLBCL and FL.^7^

**Figure 1:**
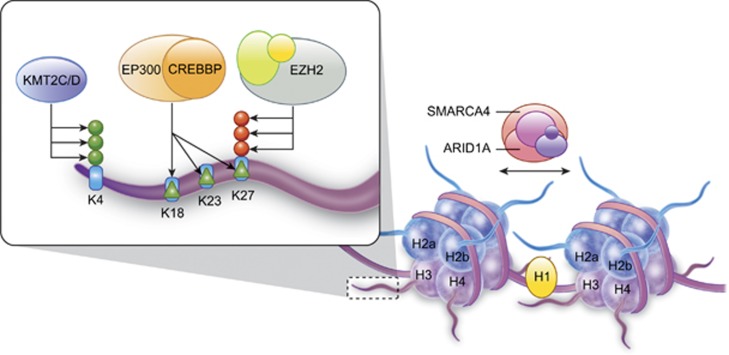
Function of chromatin modifying and organizing genes that are mutated in GCB lymphomas. A diagramatic representation shows DNA wrapped around histone octamers, consisting of histone H2a, H2b, H3 and H4, to form a nucleosome. Linker DNA between nucleosomes is bound by histone H1, and nuceolsomes are shuffled along the DNA by the SWI/SNF complex that is illustrated to include ARID1A and SMARCA4. A magnified schematic of the tail of histone H3 shows the addition of activating H3K4me3 (green circles) by KMT2C/KMT2D. This promotes the addition of activating acetylation marks (green triangles) to multiple residues on the H3 tail by recruitment of the CREBBP/EP300 complex. Activating H3K4me3 and acetylation marks oppose, and are opposed by, the repressive H3K27me3 mark (red circles) that is written by the PRC2 complex that includes EZH2. Histone H1 genes (*HIST1H1B/C/D/E*), *ARID1A*, *SMARCA4*, *KMT2C*, *KMT2D*, *EP300*, *CREBBP* and *EZH2* are each recurrently mutated in >5% of one or more subtype of lymphoma that resemble germinal center B cells (Figure 2).

**Figure 2:**
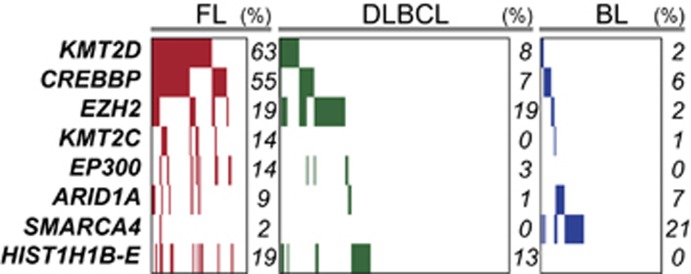
Frequency of chromatin modifying and organizing gene mutations in GCB lymphomas. Data from genome, exome and transcriptome sequencing studies of FL, BL and DLBCL with sufficient data quality^9, 10, 11, 12, 13, 14, 15, 16, 17, 18^ are summarized. Individual tumors are represented in columns and genes in rows. Colored bars indicate the presence of a somatic mutation and the percentage of tumors with mutations each gene are annotated to the right for each disease. Mutations of chromatin modifying and organizing genes are found in 84% (54/64) of FL tumors, 40% (62/155) of DLBCL tumors and 35% (29/82) of BL tumors. It should be noted, however, that DLBCL tumor are not divided by cell of origin subtypes within this diagram, and the majority of these mutations likely stratify within the GCB-like subtype. FLs are characterized by high frequencies of *CREBBP* and *KMT2D* mutations, DLBCLs are characterized by frequent mutations of HISTH1 family genes and EZH2, and BLs are characterized by high frequencies of SWI/SNF component (ARID1A, SMARCA4) mutations.

Recently, the mutation of genes encoding chromatin modifiers and organizers has emerged as a central hallmark of B-cell lymphoma, particularly those aligning with the GCB stage of differentiation. Chromatin is a complex structure of DNA and histone proteins, with each nucleosome consisting 146 bp of DNA coiled around a histone octamer (Figure 1). Chromatin can be modified by covalent modifications of histone proteins and DNA (that is, epigenetic modifications), or by ATP-dependent mobilization of nucleosomes. These processes regulate the formation or dissociation of higher order chromatin structures that can limit or promote the accessibility of DNA to transcription factors and DNA repair enzymes.^8^ Post-translational modifications of histones can induces these changes in two ways; (i) modifications can be recognized by ‘readers' that can themselves recruit additional factors including other chromatin-modifying enzymes, and (ii) acetylation and phosphorylation can act directly on chromatin structure by reducing the positive charge of histones and altering their association with negatively charged DNA. The balance between different post-translational modifications, the organization of nucleosomes and the cross talk within and between these factors can determine whether chromatin is present within an active euchromatin state or an inactive heterochromatin state. Among other marks, the formation of condensed inactive heterochromatin is associated with loss of histone H3 lysine 4 (H3K4) trimethylation, deacetylation of histones H3 and H4, trimethylation of histone 3 lysine 27 (H3K27) and dense packaging of nucleosomes that culminate in hypermethylation of promoter CpG-islands and inactivation of transcription. Here, we review the mutations of chromatin modifiers and organizers that have been found to occur in ⩾5% of one of the subtypes of non-Hodgkin's lymphoma that align with a GCB-cell stage of differentiation^7, 9, 10, 11, 12, 13, 14, 15, 16, 17, 18^ (Figure 2) and discuss how these may affect disease biology.

## Histone lysine methylation

Histone lysine methylation can occur at residues 4, 9, 27, 36 and 79 of histone H3 and residue 20 of histone H4.^19^ The locations of these modifications and the degree of methyation (that is, mono-methylation (me1), di-methylation (me2) or trimethylation (me3)) can be associated with either an active euchromatin or inactive heterochromatin state. For example, H3K4 methylation is usually associated with active transcription, but H3K4me3 is commonly localized around promoter regions whereas H3K4me1 is localized around enhancer regions. In contrast to H3K4me3, the trimethylation of lysine 27 on histone H3 (H3K27me3) mark is associated with transcriptional repression. In addition, the presence of both H3K4me3 and H3K27me3 marks are associated with a ‘poised' state that will become inactive or active following removal of either of the respective marks by histone demethylases. Unlike acetylation, histone methylation does not have a direct effect on chromatin structure. The effects are mediated by ‘reader' proteins that contain a methyl-binding domain and have a remarkable degree of specificity in recognizing unique histone modifications. This allows the recruitment of a variety of proteins, including other chromatin-modifying enzymes that promote transcriptional activation/repression and contribute to feed-forward loops and cross talk between different epigenetic marks. As a result, histone lysine methylation is a dynamic process that can encode a variety of chromatin states (reviewed by Black *et al.*^20^).

### H3K4 methylation

The *KMT2D* gene (alias, *MLL2*) is one of four members in the mixed lineage leukemia (MLL) family of proteins that have a role in H3K4 methylation. The first gene in this family, KMT2A (alias, MLL1), was discovered as a consequence of its translocation in the majority of lymphoid and myeloid leukemias arising in infants.^21^ These translocations primarily create gene fusions that interrupt the catalytic SET domain that mediates H3K4 methylation. This indicates that the oncogenic role of MLL translocations is not via enhanced H3K4 methylation, but through the recruitment of secondary factors in tandem with the fusion partner.^21^ Although there is redundancy between MLL family members with respect to their ability to add the H3K4 methylation mark to active promoters,^20^ recent findings suggest that *KMT2D* is unique in its ability to add this mark to bivalent promoters.^21^ However, KMT2D is also bound to active promoters that do not require its presence for their expression. These include the promoters of genes encoding the interleukin-7 cytokine and its receptor, which are important for early B-cell development.^22^

Germ-line mutations of *KMT2D* are associated with Kabuki syndrome, an autosomal dominant disease characterized in part by immunological defects, but without a significant predisposition to lymphomas.^23^ Somatic mutations of *KMT2D* are found in the majority of FL^9, 10, 12, 14, 16^ and less commonly in GCB-like DLBCL.^12, 13, 15^ These mutations are most commonly small deletions creating frameshifts or nucleotide variants that introduce premature stop codons, indicating that they induce a loss of function. The role of KMT2D in regulating H3K4 methylation of bivalent promoters^24^ suggests that the preferential mutation of this family member, particularly in FL, may function by changing the abundance or distribution of these transcriptionally ‘poised' states. In addition, the polycomb repressor complex 2 (discussed below) is unable to methylate H3K27 if H3K4 is trimethylated on the same histone tail,^25^ so mutations of *KMT2D* may also result in altered H3K27me3 abundance or distribution. The H3K4me3 mark is also recognized by ‘reader' proteins such as the product of the *ING1* gene, which is deleted in approximately one-third of GCB-like DLBCL.^26^ The isoforms of ING1 have multiple roles, including recruiting the CREBBP/EP300 histone acetyltransferase (HAT) complex,^27^ promoting DNA repair by linking proliferating cell nuclear antigen with EP300, and promoting p53-mediated apoptosis by linking the NuA4 complex with p53.^28^ Altered H3K4 methylation via *KMT2D* mutation and deletion of the reader protein *ING1* may therefore be alternative mechanisms for altering histone acetylation, DNA repair and p53-mediated apoptosis.

Another member of this gene family, *KMT2C* (alias, *MLL3*), is also found to be mutated at a lower frequency in FL and DLBCL,^10, 17^ providing some suggestion of functional redundancy in lymphomagenesis. Given that the majority of *KMT2D* mutations are heterozygous^10^ and there is a degree of functional redundancy within this large family of genes, an important outstanding question is whether mutation of a single *KMT2D* allele is sufficient to affect the level H3K4 methylation. To date, there has been no functional evidence to show the effect of *KMT2D* mutation on H3K4 methylation or to suggest the mechanism by which these mutations promote lymphomagenesis. This will be an important and interesting subject to be addressed by future studies.

### H3K27 methylation

EZH2 functions as a histone methyltransferase as a part of the polycomb repressor complex 2, and catalyzes the trimethylation of H3K27. This H3K27me3 mark is associated with transcriptional silencing and results in the repression of a large number of genes, including the cell cycle inhibitors encoded by the *CDKN1A* and *CDKN2A/B* genes.^29, 30^ Components of the polycomb repressor complex 2 are highly expressed in germinal centers,^31^ and the *EZH2* gene is required for germinal center formation in mice^29^ indicating that they have a role in normal GCB cellular physiology.

Mutation of *EZH2* was the first of chromatin-modifying gene alteration to be described in FL and DLBCL.^11^ In contrast to the inactivating mutations that are spread across the gene in myeloid and T-cell malignancies,^32, 33^ mutations of *EZH2* in B-cell lymphoma are localized to a ‘hotspot'. The majority of these nucleotide variants cause a single amino acid substitution of the tyrosine residue at position 641^11, 34^ and a minority affect alanine 677.^35^ In the presence of a wild-type allele that efficiently adds the first and second methyl group to H3K27, activating mutants of EZH2 show enhanced activity toward addition of the third methyl group and thereby promotes the repressive H3K27me3 epigenetic state.^36, 37^ This was recently shown to result in lymphoid hyperplasia in a mouse model harboring a tyrosine 641 mutation, but in isolation from other oncogenic events, this was insufficient to drive overt lymphoma.^29^ In addition to its role as a histone methyltransferase, EZH2 can monomethylate RORα, a DNA damage inducible protein that promotes p53 activity and apoptosis.^38^ This EZH2-mediated methylation is recognized by an E3 ubquitin ligase complex that targets RORα for degradation, thereby implicating EZH2 in the regulation of DNA damage-induced p53 activity.^38^ In addition, a proportion of EZH2 protein is localized within the cytosol where it associates with and methylates VAV1, thereby regulating actin polymerization and cell migration.^39^ However, the effect of *EZH2* hotspot mutations on its ability to methylate RORα and VAV1 and differentially regulate the activity of these non-histone proteins has not yet been investigated.

Activating *EZH2* mutations have gained further attention recently because of the development of small molecule inhibitors^40, 41, 42^ that show a high specificity for EZH2. These inhibitors decrease the abundance of di- and trimethylated H3K27 and impair the growth of lymphoma cell lines carrying *EZH2* mutations. Together, these results suggest that *EZH2* mutations may be an ‘actionable' mutation that can be targeted clinically. However, these events commonly arise as late events during disease evolution^9, 10, 14^ and are subclonal,^10^ bringing into question the degree of clinical efficacy that these inhibitors may have.

## Histone acetylation

The acetylation of histone lysine residues is a dynamic process regulated by the balance between the activity of acetyltransferases and deacetylases. Acetylation neutralizes the positive charge of lysine resides and weakens its interaction with negatively charged DNA, thereby conferring a more open chromatin structure and allowing active transcription. Two interacting HAT genes, *CREBBP* and *EP300*, are recurrently mutated in B-cell lymphoma and are most prevalent in subtypes that align with the GCB-cell stage of differentiation. The products of these genes are involved in diverse cellular processes including transcriptional activation, cell cycle progression, p53 activity, DNA repair and apoptosis.^43^ Conditional knockout of these genes within the B-cell compartment of mice revealed that loss of each gene individually had little effect on B-cell development, but loss of both genes led to a marked ablation of peripheral B cells.^44^ In humans, germ-line mutations of these genes are associated with Rubinstein–Taybi syndrome, an autosomal dominant disorder characterized by physical abnormalities and mental retardation, and associated with increased predisposition to lymphoma.^45^

*CREBBP* is targeted by inactivating mutations and deletions in FL, BL and GCB-like DLBCL.^10, 13, 15, 18^ CREBBP associates with EP300, which is itself also mutated at a lower frequency in FL and DLBCL.^10, 15^ The CREBBP/EP300 complex acts to acetylate multiple lysine residues upon all four histones,^43^ suggesting that their mutation may have broad effects on cellular phenotypes. Somatic mutations of the *CREBBP* cluster within the substrate-binding pocket of the acetyltransferase domain^10, 46^ and have been shown to decrease affinity for their substrate, acetyl-coA, resulting in a net reduction in the H3K18 acetylation mark.^15, 46^ An important target for CREBBP-mediated histone acetylation in antigen-presenting cells, including B cells, are the MHC class II genes. CREBBP is recruited to these genes by the master regulator of MHC class II gene expression, class II transactivator (CIITA), and acetylates chromatin at their promoters to activate expression.^47, 48^ Dominant-negative isoforms of CREBBP induce a 10-fold decrease in MHC class II expression in B-cell lines,^49^ and somatic mutations of *CREBBP* in FL are associated with a similar magnitude of decrease in MHC class II expression on primary tumor cells.^10^ This results in decreased T-cell proliferation and reduced numbers of T cells within *CREBBP*-mutant FL tumors, highlighting immune evasion as a key mechanism of lymphomagenesis associated with these mutations. However, the broader patterns of altered histone acetylation associated with *CREBBP* mutations remain to be defined.

In addition to its role in histone acetylation, CREBBP also acetylates the products of other genes that are themselves targeted by somatic alterations in B-cell lymphoma such as *TP53, BCL6* and *FOXO1*. The *TP53* gene is a well-defined tumor suppressor gene and is mutated and targeted by DNA copy number loss at a low frequency in a range of B-cell lymphomas. Acetylation of the *TP53* gene product by CREBBP and EP300 promotes its activity. This activation allows TP53 to recruit another HAT complex (NuA4) and activate expression of its target genes via histone H4 hyperacetylation.^28^ The *BCL6* gene encodes a transcription factor that regulates germinal center development and is targeted by genetic translocations and DNA copy number gains in DLBCL.^50, 51^ The activity of BCL6 is repressed via acetylation by CREBBP, and mutations of *CREBBP* have been linked with decreased BCL6 acetylation and increased activity.^15^ BCL6 itself also regulates chromatin modification via the recruitment of histone deacetylase complexes,^52, 53^ and the epigenetic modifications imparted by BCL6 may be sufficient for transformation even in the absence of its continued expression.^51^ FOXO1 is a PI3K-regulated transcriptional repressor that is mutated in DLBCL.^54^ Phosphorylation of FOXO1 by AKT as a result of B-cell receptor signaling has an essential role in mature B-cell survival^55^ and leads to its nuclear export, resulting in the inactivation of target genes that suppress proliferation and other key processes.^56^ Lysine acetylation within the DNA-binding motif of FOXO1 by CREBBP interferes with its DNA-binding activity and increases its sensitivity to phosphorylation, thereby contributing to its negative regulation.^57^ The activity of CREBBP to acetylate both histone and non-histone proteins that themselves regulate epigenetic and transcriptional programs suggest that inactivating mutations of this gene likely have broad phenotypic consequences at the epigenetic level, and other effects that extend beyond epigenetic programming.

In addition to *TP53* and *BCL6*, other genes that are frequently mutated or deleted in B-cell lymphoma also have a role in recruiting chromatin-modifying enzymes. For example, the well-defined tumor suppressor gene *RB1* is mutated or deleted at low frequency in B-cell lymphoma^58, 59, 60^ and recruits histone deacetylases to repress transcription of E2F target genes.^61^ Recent high-throughput sequencing studies have also identified recurrent mutations of two MEF2 family member genes, *MEF2B* and *MEF2C*, in DLBCL and FL.^12, 62, 63^ These transcription factors recruit HATs and histone deacetylases, indicating that their mutation may thereby alter the balance of histone acetylation.^64^

The somatic alteration of HATs, as well as the alteration of genes that recruit HATs and/or histone deacetylases, point to a broad deregulation of histone acetylation in B-cell lymphoma that currently remains unmapped. A potential avenue for therapeutic intervention toward deregulated histone acetylation is through the use of histone deacetylase inhibitors. Inhibitors such as Vorinostat have shown some efficacy in phase II clinical trials of relapsed/refractory FL, the disease in which *CREBBP* and *EP300* mutations are most prevalent.^65, 66^ However, a recent study that interrogated *CREBBP* and *EP300* mutation status within the bounds of a phase II trial found no significant difference in the change in tumor size between those patients with these mutations compared with patients with wild-type genes.^65^ This suggests that *CREBBP/EP300* mutations may not be ‘actionable' through the use of histone deacetylase inhibitors, and that alternative avenues for targeting these mutations need to be defined. Delineation of the precise mechanism(s) by which *CREBBP* and *EP300* mutations contribute to lymphomagenesis will be a complex task, but remains an important undefined step in understanding lymphoma pathobiology.

## Higher order chromatin structure

The positioning of nucleosomes along the DNA strand and the organization of nucleosomes into higher order chromatin structures is a dynamic process involving multiple protein complexes and non-coding RNAs, and has a crucial functional role in cellular physiology.^67, 68^ Recently, high-throughput sequencing studies have identified mutations in SWI/SNF complex and linker histone genes. These encode proteins that have a role in shuffling nucleosomes and promoting condensation of chromatin, respectively.

### Nucleosome positioning

The SWI/SNF complex is a multi-subunit complex that utilizes the energy from ATP to remodel chromatin by shuffling nucleosomes along the DNA.^8^ This regulates the accessibility of DNA to other proteins involved in replication and repair, and can allow the activation or the suppression of gene transcription. There are multiple subfamilies of SWI/SNF chromatin remodelers that are determined by their respective utilization of paralagous subcomponents. *ARID1A* and *SMARCA4* (alias, BRG1) associate with several other proteins to form BRG1-associated factor complexes, and are together mutated in 32.5% of BL tumors and less frequently in FL and DLBCL. Mutations of these genes are largely mutually exclusive and are commonly small deletions causing frameshifts, or nucleotide substitutions that introduce premature stop codons, indicating that they are deleterious to protein abundance/function. This implicates these genes as tumor suppressors, in line with prior observations that BRG1-associated factor complexes can inhibit cell cycle progression by repressing the activity of several E2F-repsonsive promoters via their association with *RB1.*^8, 69, 70^ ARID1A can also directly bind p53, enhance its transactivation activity and promote the expression of the cell cycle inhibitor CDKN1A.^71^ Interestingly, ARID1A also regulates cellular functions associated with B-cell biology. A genome-wide short hairpin RNA pool screen revealed that it may have a role in regulating sensitivity to Fas-mediated apoptosis, a central mechanism for clonal deletion of GCB cells.^72^ In addition, conditional knockout of *ARID1A* in B cells resulted in a relative decrease in proliferation in response to lipopolysaccharide compared with wild-type B cells.^73^ However, the degree to which the observations of SMARCA4 and ARID1A activity relate to the physiologic role of their mutations in lymphomagenesis remains undetermined. Notably, synthetic lethal screens of *ARID1A* mutant and *SMARCA4*-deficient cells has revealed that they are particularly vulnerable to interference with other paralagous SWI/SNF complex components.^74, 75^ This suggests that specific inhibitors of these components may represent a future avenue for therapeutic targeting of these mutations in lymphoma and other diseases.

### H1 linker histones

There are eight genes belonging to the H1 family of linker histones that are functionally redundant but differ in their expression patterns during development.^76^ These are thought to reside outside of the core nucleosome particle and protect inter-nucleosome ‘linker' DNA.^77^ Knockout experiments of H1 variants have revealed that eukaryotic cells can survive in the absence of these proteins and individual variants do not significantly alter cellular phenotypes.^78, 79, 80^ H1 proteins have a role in chromatin condensation and may function by recruiting DNA methyltransferases (DNMT1 and DNTM3A) and inhibiting methylation of H3K4.^81^ In addition, these proteins interact with the polycomb repressor complex 2, and oligonucleosomes that are assembled with H1 are better substrates for EZH2 than mononucleosomes that lack H1.^82^ Together these observations suggest that somatic alteration of histone H1 genes may potentially result in altered nucleosome packing, as well as affecting DNA and/or histone methylation. Mutations of the H1 family member gene *HIST1H1C* were first described by Morin *et al.*,^12^ and mutations within this and other genes in this family (most prevalently *HIST1H1D* and *HIST1H1E*) have been observed in multiple subsequent studies.^10, 13, 62, 63^ Although there has been some suggestion that these mutations result in decreased association with DNTM3A,^62^ it remains to be determined what the epigenetic consequences of these mutations may be, or whether they confer any measurable phenotype at all given the degree of functional redundancy between this large family of proteins.

## Discussion

The mutation of chromatin-modifying genes is likely to have a broad impact of the cellular phenotypes of B-cell lymphomas. However, despite the clearly important role for these events in lymphomagenesis, the exact mechanisms by which they promote malignant transformation remains largely undefined. An insight to this has been provided by high-throughput sequencing studies that have shown that there is a remarkable preference for specific mutations within B-cell malignancies corresponding to discrete stages of differentiation. For example, most chromatin-modifying gene mutations show the greatest recurrence frequencies in FL tumors; a malignancy that aligns with the GCB stage of differentiation.^9, 10, 14, 16^ In line with this, the most frequent chromatin modifier mutations in DLBCL (*EZH2*, *MLL2*, *CREBBP* and *EP300*) are largely restricted to a subtype of tumors that also aligns with the GCB differentiation state, and are absent from tumors that align with the later stages of differentiation (ABC-like subtype).^12^ Multiple myeloma, a malignancy aligning with the plasma cell stage of differentiation, is also devoid of these mutations, but instead possesses translocations and mutations of the H3K36 methyltransferase *WHSC1* and mutations of the H3K27 demethylase *KDM6A* (alias, *UTX*).^83, 84^ *KDM6A* mutations are also found in a precursor B-cell malignancy, B-cell acute lymphoblastic leukemia and relapses of this disease also acquire *CREBBP* mutations.^46, 85^ These patterns of representation for chromatin-modifying gene mutations among B-cell malignancies aligned with discrete differentiation states suggest that these mutations may either (i) have effects that are only oncogenic within specific cellular contexts or (ii) have roles in stalling differentiation at specific states. A recent investigation into the role of *EZH2* suggests that the latter may apply; *EZH2* hotspot mutation promotes the accumulation of GCB cells^29^ and EHZ2 inhibition promotes transition from a GCB to a memory B-cell transcriptional signature.^42^ However, these mutations are often acquired as late events in the evolution FL and may, therefore, occur secondarily to stalled differentiation.^9, 10^ The precise mechanism(s) by which mutations in chromatin modifiers promote lymphomagenesis and become associated with B-cell differentiation states are, therefore, still uncertain. Future studies that identify the role of wild-type chromatin-modifying genes in normal B-cell development, and elucidate the mechanisms by which somatic mutations of these genes drive transformation, will therefore be important for advancing our understanding of normal and malignant GCB-cell biology and in advancing therapy for lymphoma.
